# Increased injury severity and hospitalization rates following crashes with e-bikes versus conventional bicycles: an observational cohort study from a regional level II trauma center in Switzerland

**DOI:** 10.1186/s13037-022-00318-9

**Published:** 2022-03-05

**Authors:** Till Berk, Sascha Halvachizadeh, Johannnes Backup, Yannik Kalbas, Thomas Rauer, Ralph Zettl, Hans-Christoph Pape, Florian Hess, Jo Ellen Welter

**Affiliations:** 1 Department of Trauma Surgery, University Hospital Zurich, Rämistrasse 100, 8091 Zurich, Switzerland; 2 Division of Traumatology, University Hospital Zurich, Rämistrasse 100, 8091 Zurich, Switzerland; 3 Department of Trauma and Orthopedic Surgery, Berufsgenossenschaftliche Unfallklinik Frankfurt am Main, Friedberger Landstraße 430, 60389 Frankfurt am Main, Germany; 4 Cantonal Hospital Frauenfeld, Pfaffenholzstrasse 4, 8501 Frauenfeld, Switzerland

**Keywords:** Electric bicycle, E-bike, Pedelec, Bicycle crash, Injury severity, Injury severity score

## Abstract

**Background:**

As electric bicycles (e-bikes) become increasingly popular, reports of injuries associated with e-bike usage are also rising. Patterns, characteristics, and severity of injuries following e-bike crashes need further investigation, particularly in contrast to injuries from conventional bicycle crashes.

**Methods:**

This prospective observational study included 82 patients treated at a Level II trauma center for injuries resulting from an electric or conventional bicycle crash. Data were collected over one year (05.09.2017–19.09.2018) during in- and outpatient visits. A study-specific case report form was used to identify the bicycle type, cycling behavior (e.g., use of a helmet, safety gear, alcohol), and circumstances of the crash (e.g., road conditions, speed, cause of the incident, time of day, season). Additional information about patient demographics, treatment, and injury characteristics, such as the Injury Severity Score (ISS) and body region injured, were documented. Results were analyzed using chi-square, Fisher’s exact, or Wilcoxon tests. Simple logistic or linear regression models were used to estimate associations.

**Results:**

Of the 82 patients, 56 (67%) were riding a conventional bike and 27 (33%) were using an e-bike. Most incidents were either single-bicycle crashes (66%) or automobile collisions (26%), with no notable difference in prevalence rates between groups. Although a higher proportion of conventional bikers were male (67% vs. 48%), the difference was not significant. E-bikers were older (median 60 years (IQR 44–70) vs. 45 years (IQR 32–62); *p* = 0.008), were hospitalized more often (48% vs. 24%, *p* = 0.025), and had worse ISS (median 3 (IQR 2–4) vs. 1 (IQR 1–3), *p* < 0.001), respectively. Body regions most affected were the extremities (78%) and external/skin (46%), and these were distributed similarly in both groups. Concomitant injury patterns of the thorax/chest with external/skin were higher among e-bikers (p < 0.001). When we controlled for the difference in the median age of the two groups, only the injury severity score of e-bikers remained significantly worse.

**Conclusions:**

Hospitalization and chest trauma rates were higher among e-bikers. After controlling for the older age of this group, the severity of their injuries remained worse than in conventional cyclists. Initial clinical assessments at trauma units should include an evaluation of the thorax/chest, particularly among elderly e-bikers.

**Level of evidence:**

Level III.

## Background

Electric-assisted bicycles (e-bikes) are gaining popularity in Switzerland. In terms of overall sales, 2020 was the most profitable year in the Swiss bicycle industry’s history, with one-third of sales being e-bikes [1, 2]. This increasing trend in e-bike usage is also happening in other parts of the world - an estimated 466 million e-bikes were in use in 2016 [1].

E-bikes have a small electric motor that can be integrated as a hub motor (at the front or rear wheel) or a mid-drive motor (into the crank near the pedals). Although the types (class categories) of e-bikes vary, the maximum speed (with pedal assistance) ranges from 32 to 45 km/hour [3], and they are approximately 40–50% heavier than traditional bikes (18–22 kg vs. 12–13 kg). Despite its convenience, particularly for elderly riders, the electric-powered bicycle presents a potential safety challenge for public health. Several studies have found an increase in non-fatal and fatal injuries related to e-bike crashes [4–8]. Conversely, other studies that assessed injury severity did not find that e-bikers had significantly more crashes or severe injuries [9–12]. Further investigations on injury patterns and characteristics of these road traffic incidents are needed [1, 12].

Our study aimed to describe the circumstances of crashes occurring while riding a conventional or electric bicycle that resulted in injuries warranting either in- or outpatient care. Furthermore, we aimed to identify and compare the cyclists’ behavior, injury patterns (severity, type, and treatment required), and patient characteristics potentially associated with a higher risk of morbidity.

## Methods

### Study design and participants

This observational study was approved by the local ethics committee (Canton Thurgau; Nr. 2017–01946), and written consent was obtained from all participants. The study was conducted in accordance with the Declaration of Helsinki. It was carried out over one year (05.09.2017–17.09.2018) and included bicycle crash patients treated at in- or outpatient units of a Level II trauma center in the northeastern region of Switzerland. The trauma center is part of the Thurgau cantonal hospital system serving a population of approximately 273,800 inhabitants (Swiss Federal Statistical Office, 2017). A predominantly agricultural region, the elevation of the canton ranges from 368 to 991 m above sea level.

We enrolled patients over an entire calendar year to detect seasonal differences. German-speaking patients 16 years and older who crashed on either a conventional or electric bicycle were assessed for inclusion. Any patient with a genetic disorder affecting the musculoskeletal system, a cognitive disorder, or an oncologic disease was excluded. In addition, we excluded pedestrians hit by a bicycle or anyone involved in a motorcycle crash.

We assessed for inclusion 105 cyclists who sought medical attention at our center. Twenty-one were excluded because they were under 16 years of age and two refused to participate. Therefore, a total of 82 patients were included in our analysis, of which 61% were male (*n* = 50) and 39% were female (*n* = 32).

### Outcomes

All patients were treated according to hospital guidelines. Data were collected at the initial point of care only; no additional follow-up information was obtained as most patients sought follow-up care, when needed, from their primary care physicians. In addition to documenting the patient’s medical history, a questionnaire (paper) was administered to the patient by a trained staff member. Variables collected on this form were as follows: the cause of injury (e.g., single-bicycle crash, collision with a pedestrian or a motor vehicle), environmental circumstances (e.g., weather/road conditions, time of day/year), the use of safety clothing/gear, use of drugs or alcohol at the time of the incident, and the estimated speed at the time of the crash. As part of the routine medical exam, the following information was documented in the patient’s medical records: age, gender, date of the incident/visit, need for and length of hospitalization, treatment required, and medical diagnosis classifications (according to the International Classification of Diseases, 9th revision, Clinical Modification – ICD-9-CM).

Associated injuries were documented for six body regions (head/neck [including cervical spine], face [including facial skeleton, nose, mouth eyes, and ears] thorax/chest [including thoracic spine and diaphragm], abdomen or pelvic contents [including abdominal organs and lumbar spine], extremities and pelvic girdle [including pelvic skeleton], external/skin [including lacerations, contusions, abrasions]). Patient injuries were further categorized based on their injury severity score (ISS) [13], with scores ranging from 0 to 75 (best to worst). Serious injury was defined as ISS ≥ 9 and severe injury was ISS ≥ 16.

### Statistical analysis

Continuous variables were summarized based on normality tests (Shapiro-Wilks) - mean with standard deviation (±SD) or median (interquartile range IQR). Categorical variables were presented as frequency and percentages, and comparisons between groups were done with either chi-square or Fisher’s exact tests. Wilcoxon rank-sum tests were used for comparing continuous variables between groups. For binary outcomes, we used simple logistic regression to test the association of an independent variable, and for continuous dependent variables we tested associations using simple linear regression. The significance level was set at *p* < 0.05. All tests were conducted in Stata version 15.1 (Stata Corporation, College Station, TX, USA). We used a convenience sample of all consecutive patients treated over one calendar year.

## Results

After applying the exclusion criteria to a cohort of 105 consecutive patients who had a bicycle accident during the study period, a total of 82 patients were included in this study. Of these, 55 patients (67%) were riding a conventional bicycle at the time of the incident and 27 (33%) were operating an e-bike. The median age of the entire cohort was 51 years (IQR 33–64), with a range of 16 to 84 years. Table 1 presents patient demographics, cycling behavior, and injury-related factors grouped according to bicycle type. The most notable difference was that the e-bikers were significantly older than the conventional bikers.

**Table 1:** Characteristics of patients, cycling behavior, and accident-related factors according to bicycle type

	Conventional bike (***n*** = 55)	Electric bike (***n*** = 27)	***p***-value
Male	37 (67%)	13 (48%)	0.095
Age at time of accident (years)^a^	45 (32–62)	60 (44–70)	**0.008**
Helmet use	38 (69%)	17 (63%)	0.825
Alcohol or drug use	8 (15%)	5 (19%)	0.434
Prescription drug use	12 (22%)	11 (41%)	0.127
Frequency of rides per week	4 (2–7)	5 (4–7)	0.184
Estimated number of hours riding per week^a^	3.5 (1–7)	4 (2–8)	0.337
Cause of accident			0.227
Single-bicycle (fell/thrown from bicycle)	36 (65%)	18 (66%)	
Collision with motor vehicle	13 (24%)	8 (30%)	
Collision with pedestrian	0	1 (4%)	
Other (e.g., collision with stationary object)	6 (7.3%)	0	
Estimated speed at time of accident (km/hour)^a^	16 (10–20)	15 (10–23)	0.959
Season			0.436
January–March	4 (7%)	0	
April–June	25 (45%)	10 (37%)	
July–September	19 (35%)	12 (44%)	
October–December	7 (13%)	5 (19%)	
Wet or snowy road conditions	6 (11%)	2 (7%)	0.999
Time of crash			0.273
00:01–00:06:00	4 (7%)	1 (4%)	
06:01–00:12:00	16 (29%)	10 (37%)	
12:01–00:18:00	22 (40%)	14 (52%)	
18:01–00:00:00	13 (24%)	2 (7%)	

^a^Data presented as median (interquartile range) were tested with Wilcoxon ranked sum; Fisher’s exact or chi-square tests used for categorical data; significance level set at < 0.05

### Hospitalization rates and injury severity

There was a significantly higher need for hospitalization among the e-bikers than conventional bikers (13 (48%) vs. 13 (24%), *p* = 0.025). However, while the median length of the hospitalization was longer among the e-bikers, it was not statistically significant (e-bike 3 days (IQR 2–4) vs. conventional bike 1 day (IQR 1–3), *p* = 0.133). Likewise, the need for surgical intervention was higher among e-bikers but not significantly different (e-bike 11 (41%) vs. conventional bike 16 (29%), *p* = 0.291).

Regarding the severity of the injuries sustained, the median ISS was worse among the e-bikers than conventional bikers (3 (IQR 2–4) vs. 1 (IQR 1–2), *p* < 0.001). Nevertheless, the median score of the entire cohort was relatively low (2 (IQR1–4); range 1–16). Only one patient in the conventional bike group had a score above 9, and one patient in the e-bike group had a score of 16. Table 2 presents the distribution of injuries by ISS body region. The majority of the cohort (*n* = 50, 61%) sustained injuries to more than one body part; however, no significant difference was found between the groups (e-bikers 67% vs. conventional bikers 58%, *p* = 0.483). The body region most frequently injured in the entire cohort was extremities (78%), followed by external injuries (skin) (46%).

**Table 2:** Distribution of injuries by Injury Severity Score (ISS) body region according to bicycle type

ISS body region	Total (***n*** = 82)	Conventional bike (*n* = 55)	Electric bike (*n* = 27)	*p*-value^a^
Head / neck	18 (22%)	12 (22%)	6 (22%)	0.999
Face	14 (17%)	10 (18%)	4 (15%)	0.999
Thorax / chest	20 (24%)	10 (18%)	10 (37%)	0.099
Abdomen	5 (6%)	4 (7%)	1 (4%)	0.099
Extremities	64 (78%)	44 (80%)	20 (74%)	0.578
External (skin)	38 (46%)	21 (38%)	17 (63%)	0.058
1 body region injured	32 (39%)	23 (42%)	9 (33%)	0.483
2 body regions injured	32 (39%)	22 (40%)	10 (37%)	0.999
≥3 body regions injured	18 (22%)	10 (18%)	8 (30%)	0.266

^a^ Fisher’s exact test; significance level set at < 0.05

When analyzing concomitant injuries, we found that thorax/chest injuries together with external/skin injuries were significantly higher among the e-bikers (22% vs. none, *p* < 0.001). No other combination of injury types was significantly different between the groups. The most frequent concomitant injury patterns in the conventional bike group were as follows: extremity with external/skin (38%, *n* = 21), head/neck with face (13%, *n* = 7), and head/neck with thorax/chest (11%, *n* = 6). In the e-bike group, the most common patterns were the following: extremity with external/skin (63%, *n* = 17), thorax/chest with external/skin (22%, n = 6), and head/neck with external/skin (18.5%, *n* = 5).

Results from simple logistic regression with the outcome (y) *need for hospitalization* and predictor (x) *age* indicated an association [OR = 1.01, 95%CI = 1.01–1.06, *p* = 0.039]. However, when we did simple linear regression with ISS as the outcome and used the same predictor variable, no association was detected [β = 0.014, F(1,78) = 2.44, *p* = 0.122, R^2^ = 0.0304], indicating that the older age of the e-bike group was unlikely to confound our findings that more severe injuries occurred in patients who had operated an e-bike.

## Discussion

Our study included data from crashes while riding a conventional or electric bicycle, resulting in an injury warranting either in- or outpatient care at a Level II trauma center. We identified and compared cyclists’ behavior, injury patterns (severity, type, and treatment required), and patient characteristics of these two groups. Overall, the severity of injuries was low, and the cause of most incidents was a single bicycle crash. The people riding an e-bike were older, had a higher rate of injury to multiple body parts, were more likely to be admitted to the hospital, and had a higher rate of concomitant chest injuries. After adjusting for the age difference between groups, only the severity of the injuries was significantly worse among the e-bikers.

Although several earlier studies reported similar findings concerning higher injury severity among e-bikers [9, 11, 14–17], little to no difference in the severity of injuries between the groups was detected in other studies [10, 11, 18, 19]. A positive correlation has been observed between increasing age and injury severity in e-bikers [11, 20–22]. Furthermore, Verstappen et al. and Spörri et al. reported that e-bikers were not only older but had more comorbidities than conventional bikers [17, 18]. In our study, the type of care provided at our institution (Level II trauma center) limited the extent and severity of the cases included in the analysis. More severely injured patients, including those with traumatic brain injuries (TBI), would have been diverted to higher-level facilities for specialized care. Although we may have missed opportunities to assess more severe injury types such as TBI, our comparison had the advantage of concentrating on a subset of mild-to-moderate injuries.

The literature about injury distribution among e-bikers is somewhat limited and often based on small case series [4, 6, 11, 12, 23]. Verstappen et al. also found a higher rate of chest and soft tissue injuries in e-bikers [18]. Further investigations into plausible causes of chest and thoracic trauma are warranted. Although only speculative, this finding in our cohort may be associated with a slower reaction time of older e-bikers. Regardless of the cause, protective clothing may help prevent or lessen the severity of injuries to the chest. We also found high rates of injuries to the skin, which could also be attributed to the older age of the e-biker group; however, other studies found significantly higher injury rates to the upper extremities only [24, 25].

A multitude of factors may be contributing to the types of injury patterns of e-bikers. Although the leading cause of the incidents (single bicycle crash) was the same for our two bicycle groups, the weight of the electronic bicycle with the heavy battery [26] may play a role in the extent of the injury. We did not ask patients about their perceived ability or comfort in operating an e-bike. However, reduced muscle mass associated with older age [27] can negatively impact a rider’s strength and ability to brace for impact in the event of a crash. Future studies should assess whether riders’ experience level is more likely to lead to severe injuries than their physical limitations. Given the potentially high speed and increased weight of an e-bike, proof of one’s ability to maneuver and operate an electric-powered bicycle through licensure may be recommended.

Our study had some limitations. First, we collected data from a convenience sample of consecutive patients seen at a Level II trauma center over one calendar year. No power analysis was done to determine a sample size large enough to detect differences in the groups. Consequently, we had an imbalanced distribution in our cohort. Nevertheless, the relatively small number in the e-bike group was proportional to e-bike usage in the geographical region and time the study was carried out. Second, although we used prescription drug use as a proxy for health status at the time of the incident, other types of baseline indicators could have been documented and compared, such as the ASA score, bone density (osteoporosis), comorbidities, and BMI. Lastly, we could not verify the speed at the time of the crash, which could have been under- or overestimated by the cyclists. Interestingly, the conventional bikers reported a slightly higher median speed than the e-bikers. Although the likelihood of having a speedometer is greater on e-bikes, the rider may not be actively monitoring their velocity. The perception of speed may have been affected by factors such as 1) varying amounts of effort or strain needed for the bicycle types, 2) whether the bicycle was used for exercise or transport purposes, and 3) the experience level of the cyclist. Regardless, the hypothesis that e-bikers are at increased risk of severe injury due to higher speeds should be investigated further.

## Conclusions

This study contributes additional data about the type and severity of injuries from electric bicycle crashes and how these injuries compared with conventional bicycle crashes. Overall, the median ISS of our study groups were low, which reflects, in part, the target population of our trauma center (Level II). However, we found that the severity of injuries was significantly worse in the e-bike group, and particularly worrisome was the increased likelihood of trauma to the thorax/chest. As the popularity of electric bikes continues to rise in Switzerland, the safety issues associated with this mode of transport need further evaluation. More information about the causes of the incidents and the body regions most likely to be affected can be used to prevent or lessen the magnitude of future injuries. Hospitalization and chest trauma rates were higher among e-bikers. After controlling for the older age of this group, the severity of their injuries remained worse than in conventional cyclists. Initial clinical assessments at trauma units should focus on evaluation of the thorax/chest, particularly among elderly e-bikers.

## Data Availability

The datasets generated and analyzed during the current study contain patient data; therefore, they are not publicly available due to local and cantonal data protection policies.

## Abbreviations

ASA: American Society of Anesthesiology
BMI: Body mass index
ICD: International Classification of Diseases
IQR: Interquartile range
ISS: Injury Severity Score
SD: Standard deviation
TBI: Traumatic brain injury

## References

[1] Papoutsi S, Martinolli L, Braun CT, Exadaktylos AK (2014). E-bike injuries: experience from an urban emergency department-a retrospective study from Switzerland. Emerg Med Int.

[2] Schweizer Fachstelle Velo und E-Bike. Entwicklung Schweizer Fahrrad- und E-Bike-Markt, 2005 bis 2020 2020 [Available from: https://www.velosuisse.ch/wp-content/uploads/2021/03/Gesamt_2005-2020_Veloverkaufsstatistik_Schweizer_Markt.pdf.

[3] Cherry CR, MacArthur JH. E-bike safety. A review of Empirical European and North American Studies. Light Electric Vehicle Education and Research Initiative 2019. https://wsd-pfbsparkinfluence. s3. amazonaws.com/uploads/2019/10/EbikeSafety-VFinal. pdf.

[4] Du W, Yang J, Powis B, Zheng X, Ozanne-Smith J, Bilston L (2014). Epidemiological profile of hospitalised injuries among electric bicycle riders admitted to a rural hospital in Suzhou: a cross-sectional study. Injury Prev.

[5] Du W, Yang J, Powis B, Zheng X, Ozanne-Smith J, Bilston L (2013). Understanding on-road practices of electric bike riders: an observational study in a developed city of China. Accid Anal Prev.

[6] Feng Z, Raghuwanshi RP, Xu Z, Huang D, Zhang C, Jin T (2010). Electric-bicycle-related injury: a rising traffic injury burden in China. Injury Prev.

[7] Tenenbaum S, Weltsch D, Bariteau JT, Givon A, Peleg K, Thein R (2017). Orthopaedic injuries among electric bicycle users. Injury..

[8] Zhang X, Cui M, Gu Y, Stallones L, Xiang H (2015). Trends in electric bike-related injury in China, 2004–2010. Asia Pac J Public Health.

[9] Hu F, Lv D, Zhu J, Fang J (2014). Related risk factors for injury severity of e-bike and bicycle crashes in Hefei. Traffic Inj Prev.

[10] Lawinger T, Bastian T. New appearances of powered two-wheelers. An empirical in depth study of accidents with Pedelecs in the state of Baden-Württemberg. Zeitschrift fuer Verkehrssicherheit Ausgabe 2013;2:2013.

[11] Schepers JP, Fishman E, den Hertog P, Wolt KK, Schwab AL (2014). The safety of electrically assisted bicycles compared to classic bicycles. Accid Anal Prev.

[12] Weber T, Scaramuzza G, Schmitt KU (2014). Evaluation of e-bike accidents in Switzerland. Accid Anal Prev.

[13] Baker SP, O'Neill B. The injury severity score: an update. Journal of trauma and acute care. Surgery. 1976;16(11):882–5. 10.1097/00005373-197611000-00006 994270

[14] Capua T, Glatstein M, Hermon K, Tavor O, Scolnik D, Kusaev V, et al. A comparison of manual versus electric bicycle injuries presenting to a pediatric emergency department. Rambam Maimonides Med J. 2019;10(3). 10.5041/RMMJ.10370 PMC6649777 31335313

[15] Poos H, Lefarth TL, Harbers JS, Wendt KW, El Moumni M, Reininga IHF (2017). E-bikers are more often seriously injured in bicycle accidents: results from the Groningen bicycle accident database. Ned Tijdschr Geneeskd.

[16] Siman-Tov M, Radomislensky I, Peleg K (2017). The casualties from electric bike and motorized scooter road accidents. Traffic Inj Prev.

[17] Spörri E, Halvachizadeh S, Gamble JG, Berk T, Allemann F, Pape HC, et al. Comparison of injury patterns between electric bicycle, bicycle and motorcycle accidents. J Clin Med. 2021;10(15):3359. 10.3390/jcm10153359 PMC8347860 34362145

[18] Verstappen EMJ, Vy DT, Janzing HM, Janssen L, Vos R, Versteegen MGJ, et al. Bicycle-related injuries in the emergency department: a comparison between E-bikes and conventional bicycles: a prospective observational study. Eur J Trauma Emerg Surg. 2020;47(6):1853–60. 10.1007/s00068-020-01366-5 32306122

[19] Beratungsstelle für Unfallverhütung. Verkehrssicherheit von E-Bikes mit Schwerpunkt Alleinunfälle 2018 [Available from: https://www.bfu.ch/media/cvud2vwz/bfu_2-340-01_bfu-report-nr-75-verkehrssicherheit-von-e-bikes-mit-schwerpunkt-alleinunfaelle.pdf.

[20] Eilert-Petersson E, Schelp L (1997). An epidemiological study of bicycle-related injuries. Accid Anal Prev.

[21] Kim JK, Kim S, Ulfarsson GF, Porrello LA (2007). Bicyclist injury severities in bicycle-motor vehicle accidents. Accid Anal Prev.

[22] Kim YJ, Seo DW, Lee JH, Lee YS, Oh BJ, Lim KS (2017). Trends in the incidence and outcomes of bicycle-related injury in the emergency department: a nationwide population-based study in South Korea, 2012-2014. PLoS One.

[23] Otte D, Facius T, Mueller C (2014). Pedelecs in road traffic accidents and comparison to conventional non-motorized bicycles. VKU Verkehrsunfall und Fahrzeugtechnik.

[24] Gioffrè-Florio M, Murabito LM, Visalli C, Pergolizzi FP, Famà F (2018). Trauma in elderly patients: a study of prevalence, comorbidities and gender differences. Il Giornale di chirurgia.

[25] Palvanen M, Kannus P, Parkkari J, Pitkäjärvi T, Pasanen M, Vuori I (2000). The injury mechanisms of osteoporotic upper extremity fractures among older adults: a controlled study of 287 consecutive patients and their 108 controls. Osteoporos Int.

[26] Popovich N, Gordon E, Shao Z, Xing Y, Wang Y, Handy S (2014). Experiences of electric bicycle users in the Sacramento, California area. Travel Behav Soc.

[27] Baumgartner RN, Waters DL, Gallagher D, Morley JE, Garry PJ (1999). Predictors of skeletal muscle mass in elderly men and women. Mech Ageing Dev.

